# Do Bad Smells Cause Disease?

**Published:** 1860-04

**Authors:** 


### DO BAD SMELLS CAUSE DISEASE?

The tendency of the human mind to rest satisfied with any belief that is authoritatively asserted, is too well known to require any comment. Philosophers of all kinds are no more exempt than other people from this easy style of dealing with difficult problems. Medicine is, we think, especially chargeable with cherishing pet answers to questions that force themselves unkindly on her; and we think that the way in which she has made up her mind as to the causes of various kinds of fevers is an example of this style of cutting the Gordian knot.

Of late years, it must have struck all our readers that pig-styes, dirty pools of water, open privies, ash-heaps, etc., have been declared highly criminal, and on all occasions even adjudged guilty of producing any kind of fever or bowel complaint that may have broken out in the neighborhood. If a child happen to suffer typhus in a farmhouse, it is the mixen at the end of the barton that caused it. If an epidemic of English cholera befall a village, it is traced to the duck-pond by the road-side. If in a wealthy household the inmates are stricken with diphtheria, some open sewer close at hand, has, as a matter of course, been the cause. So accustomed are we to hear this sort of reasoning resorted to on all occasions, that one feels a little difficulty in expressing doubts as to the certainty with which the effect is unhesitatingly traced to its cause. Nevertheless, we think there is at least sufficient evidence to cause reflecting minds to pause ere they give in their adhesion to the general opinion, and thus shut their eyes to further research and inquiry. Dr. Watson, has, we know, stated it as his distinct opinion, "*that neither animal nor vegetable decomposition is sufficient to generate fever of any kind;*" and the researches of Dr. Guy, and other observers, have certainly gone some way to support that opinion.

Dr. Guy, in his very interesting contribution to the *Journal of the Statistical Society*, on the health of Nightmen, Scavengers, and Dustmen, gives us a mass of statistical facts which, it must be confessed, run counter to the generally received opinion, that foul animal or vegetable emanations are the fruitful source of disease. This class of men, without doubt, spend their days in the very midst of filth of all kinds. He says:

"In most of the laystalls or dustmen's yards, every species of refuse matter is collected and deposited—night-soil, the decomposing refuse of markets, the sweepings of narrow streets and courts, the sour-smelling grains from breweries, the surface-soil of the thoroughfares, and the ashes from the houses."

This heterogeneous mass the scavengers or "hill" people have to sort or to pass through sieves, so that the emanations arising therefrom must be brought into intimate relation with their lungs and skin. If fever and diarrhœa are so clearly traceable to the vicinity of these so-called noxious materials, surely the scavengers ought to be a poor, fever-stricken race. A medical examination, however, of this class of workmen as compared with brickmakers and bricklayers' laborers, proves that the scavenger is comparatively exempt from disease. Thus, among a number of men examined in each of the three classes, it appeared that the numbers attacked by fever were, among the scavengers, 8 per cent.; among the bricklayers' laborers, 35.5 per cent.; and among brickmakers, 21.5 per cent.

This result seems extraordinary enough; but it may be argued that these men do not live in the laystalls or dustyards, and therefore that their exemption from fever may be attributable to this; but what can be said if the master dustmen and their families, who live all their lives in the midst of these heaps of so-called fever-nests, are healthy. Dr. Guy says:

"I do not think that, whether in town or country, such another body of men (as master dustmen) could be brought together, except by selection; and it is not going too far to assert of them that, if the comparison were limited to the inhabitants of London, or our large towns, no score of selected tradesmen could be found to match the same number of scavengers brought casually together."

Unless we suppose that the scavengers get used to this so-called miasmatic atmosphere, or that after a time it no longer affects them, we cannot see how the foul emanation theory can hold water. Nature cannot work in one place differently from another. Night-soil must be just as deadly in an open yard in London as in the country. But here we have the experiment tried on a larger scale, of a whole class of men subjected to foul emanations, and yet they are far from being an unhealthy race, and are not nearly so prone to fever or bowel disease as the brickmakers' laborers.

We are far from wishing it to be understood, however, that we do not consider foul emanations as dangerous or baneful under any circumstances. In our opinion, they become noxious when much concentrated. Our houses, for instance, are built on the principle of a bell-glass; and our drains and privies, and all other impurities, if allowed to give off a deleterious miasma, most certainly do become most virulent sources of disease. But, in the open air, we think it very doubtful whether these emanations are ever the cause of injury to man.

Let us watch with Dr. McWilliam a still more gigantic experiment on the health of the Thames waterside people, which has been going on for years, and is still proceeding. The whole sewerage of two millions and a half of people, has, within the last ten years, been turned into the metropolitan stream. Year by year its waters have become more contaminated, and its smell more disgusting. It should follow that the health of the waterside community is proportionally decreasing; that febrile complaints, cholera, and diarrhœa, are alarmingly on the advance. But what is the real state of the case? Dr. McWilliam, in his Report for the year 1858, on the health of the Water Guard and Waterside Officers of Her Majesty's Customs, says:

"As respects bowel affections, in which I include diarrhœa, choleraic diarrhœa, dysentery, etc., the types of those forms of disease, which in this country noxious exhalations are commonly supposed to originate, we find the additions during the four hot months of the past year from this class of complaints 26.3 below the average of the corresponding period of the three previous years, and 73 less than those of 1857."

The quantity of putrescent animal and vegetable matter in the Thames has been going on increasing; but the illness generally attributed to the emanations arising therefrom has been decreasing! We know that many will urge that all the combustibles (if we may use the term) being thus accumulated, it only requires the match to be applied, to find epidemics raging like wildfire. But the year before last, cholera did break out on the banks of the Lea, and there died out, apparently from want of sustenance. This year, according to the *Lancet*, cholera, veritable Asiatic cholera, has been on board the Dreadnaught; yet it has not spread, and there seems no likehood of its doing so for this season at least. As Dr. McWilliam tru!y says, "It is nowhere sustained by evidence that the stench from the river or docks, however noisome, was in any way productive of disease." It is true that one waterman, in June last, was said to have died of Asiatic cholera, and that his death was ascribed to river-poison; but, as the eminent observer whom we have just quoted, correctly remarks, "it is opposed to all analogy, and to the usual order of nature, and therefore unphilosophical, to suppose that a cause so extensively diffused should have been so singularly limited in its effect."

Greatly doubting, as we do, the alleged ill effects of foul emanations in the open air upon human life, we nevertheless do not think that the crusade against filth should for one moment be relaxed. A bad smell may be no more unhealthy than a bad taste; but we should, if possible, avoid the one as much as the other. What we should, above all things, avoid, however, is the falling into the error of supposing that bad smells are the indubitable sources of many puzzling diseases, and of thus hardening our minds against investigations of the kind which were instituted a year or two ago by Dr. Barker, and which, when completely carried out, will enable us to decide what the noxious principles are which make all the difference between an unpleasant and a malarious odor.

